# Analysis of positron emission tomography hypometabolic patterns and neuropsychiatric symptoms in patients with dementia syndromes

**DOI:** 10.1111/cns.14169

**Published:** 2023-03-16

**Authors:** Jinghuan Gan, Zhihong Shi, Chuantao Zuo, Xiaobin Zhao, Shuai Liu, Yongjie Chen, Nan Zhang, Li Cai, Ruixue Cui, Lin Ai, Yi‐Hui Guan, Yong Ji

**Affiliations:** ^1^ Department of Neurology, Beijing Tiantan Hospital Capital Medical University, China National Clinical Research Center for Neurological Diseases Beijing China; ^2^ Department of Neurology, Tianjin Dementia Institute, Tianjin Key Laboratory of Cerebrovascular and Neurodegenerative Diseases Tianjin Huanhu Hospital Tianjin China; ^3^ PET Center, Huashan Hospital Fudan University Shanghai China; ^4^ Department of Nuclear Medicine, Beijing Tiantan Hospital Capital Medical University Beijing China; ^5^ Department of Epidemiology and Statistics, School of Public Health Tianjin Medical University Tianjin China; ^6^ Tianjin Key Laboratory of Environment, Nutrition and Public Health Tianjin China; ^7^ Department of Neurology General Hospital of Tianjin Medical University Tianjin China; ^8^ Department of PET‐CT Diagnostics Tianjin Medical University General Hospital Tianjin China; ^9^ Department of Nuclear Medicine, Peking Union Medical College Hospital Chinese Academy of Medical Sciences and Peking Union Medical College Beijing China

**Keywords:** cognitive impairment, fluorodeoxyglucose positron emission tomography, frontotemporal lobar degeneration, hypometabolism, neuropsychiatric symptom

## Abstract

**Aims:**

To estimate the proportions of specific hypometabolic patterns and their association with neuropsychiatric symptoms (NPS) in patients with cognitive impairment (CI).

**Methods:**

This multicenter study with 1037 consecutive patients was conducted from December 2012 to December 2019. ^18^F‐FDG PET and clinical/demographic information, NPS assessments were recorded and analyzed to explore the associations between hypometabolic patterns and clinical features by correlation analysis and multivariable logistic regression models.

**Results:**

Patients with clinical Alzheimer's disease (AD, 81.6%, 605/741) and dementia with Lewy bodies (67.9%, 19/28) mostly had AD‐pattern hypometabolism, and 76/137 (55.5%) of patients with frontotemporal lobar degeneration showed frontal and anterior temporal pattern (FT‐P) hypometabolism. Besides corticobasal degeneration, patients with behavioral variant frontotemporal dementia (36/58), semantic dementia (7/10), progressive non‐fluent aphasia (6/9), frontotemporal lobar degeneration and amyotrophic lateral sclerosis (3/5), and progressive supranuclear palsy (21/37) also mostly showed FT‐P hypometabolism. The proportion of FT‐P hypometabolism was associated with the presence of hallucinations (*R* = 0.171, *p* = 0.04), anxiety (*R* = 0.182, *p* = 0.03), and appetite and eating abnormalities (*R* = 0.200, *p* = 0.01) in AD.

**Conclusion:**

Specific hypometabolic patterns in FDG‐PET are associated with NPS and beneficial for the early identification and management of NPS in patients with CI.

## Graphical abstract

Over half of patients with AD and DLB had AD‐pattern hypometabolism. Frontotemporal lobe pattern (FT‐P) hypometabolism was common in FTLD, especially in patients with bvFTD, SD, PNFA, FTLD/ALS, and PSP. The proportion of FT‐P hypometabolism was associated with the presence of hallucinations, anxiety, and appetite and eating abnormalities in AD. Hypometabolic patterns can help to early identify and manage neuropsychiatric symptoms in cognitive impairment.

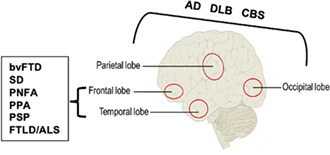

## 1 INTRODUCTION

Cognitive impairment (CI), including mild cognitive impairment (MCI) and dementia, has been a major global health problem because of the aging of the world's population. Dementia is broadly characterized by cognitive and psychological dysfunction that significantly impairs daily and social functioning. Overall, approximately more than half of dementia cases are attributable to Alzheimer's disease (AD), followed by vascular dementia (VaD), dementia with Lewy bodies (DLB), and frontotemporal lobar degeneration (FTLD). Currently, early identification, differential diagnosis, and improved therapy have become an important part of CI management.

[18F]‐Fluorodeoxyglucose positron emission tomography (18F‐FDG‐PET) is a biomarker for neuronal degeneration with good diagnostic accuracy, thus it is widely used in current clinical research and has now been recommended as a reliable tool for diagnosis in patients with CI.^1^ AD shows hypometabolism predominantly in a posterior pattern, including in the posterior temporoparietal association cortex and posterior cingulate cortex. Patients with DLB share the characteristic of a posterior pattern of hypometabolism that is seen in patients with AD and also demonstrate hypometabolism in the occipital lobe.^2^ This pattern is characterized by the “cingulate island sign,” which demonstrates a parieto‐occipital pattern of hypometabolism and relatively preserved posterior cingulate metabolism.^3^, ^4^ The metabolic abnormality in FTLD is predominant in the frontal and anterior temporal lobes, cingulate gyri, uncus, insula, and the subcortical areas, including basal ganglia and medial thalamic regions.^5^, ^6^, ^7^ AD‐pattern hypometabolism represents decreased clinical stability, with accelerated progression from amnestic MCI to AD.^8^ Moreover, longitudinal FDG‐PET data revealed that (i) greater medial temporal and posterior cingulate hypometabolism were related to memory decline; (ii) asymmetrical lateral temporal hypometabolism was related to language dysfunction; (iii) hypometabolism in the lateral parietal lobe and precuneus hypometabolism were related to visuospatial dysfunction and hallucinations; and (iv) progressive frontal lobe hypometabolism was related to executive dysregulation and depression.^9^, ^10^, ^11^ Until now, there has been a lack of studies on hypometabolic patterns in large samples of FDG‐PET in patients with CI, especially in patients with FTLD subtypes. In addition, there have been few studies that evaluate the correlation between hypometabolic patterns in neuroimaging and neuropsychiatric symptoms (NPS) in patients with dementia.

Therefore, we performed a multicenter study to estimate the proportion of the primary hypometabolic pattern in a large sample of patients with a variety of MCI and dementia syndromes, evaluate the associations between AD‐pattern (AD‐P) and frontotemporal lobe‐pattern (FT‐P) hypometabolism and NPS, and examine the apolipoprotein E (APOE) ε4 allele, amyloid‐β (Aβ), or Tau deposition on PET scans in this cohort of patients.

## 2 MATERIALS AND METHODS

The Ethics Committees of Tianjin Huanhu Hospital, Beijing Tiantan Hospital, Shanghai Huashan Hospital, Peking Union Medical College Hospital, and Tianjin Medical University General Hospital approved all research activities in this cohort study and waived informed consent because the data were pseudonymized from registers. The study followed the Strengthening the Reporting of Observational Studies in Epidemiology reporting guideline.

### 2.1 Subjects

A total of 1037 consecutive patients with CI were recruited at the PET/CT Center of Beijing Tiantan Hospital, Shanghai Huashan Hospital, the Cognitive Clinic of Tianjin Huanhu Hospital, Peking Union Medical College Hospital, and Tianjin Medical University General Hospital between December 2012 and December 2019. All participants were 40–92 years of age. The inclusion criteria were as follows: a clinical diagnosis of MCI, dementia of any type or other unclassifiable dementia (ODs), and imaging with ^18^F‐FDG PET, with or without Aβ‐PET (^11^C‐PIB PET or ^18^F‐AV45 PET), or Tau‐PET ([^18^F]PM‐PBB3) within 3 months of the initial clinical diagnosis.

For the present analyses, 382 patients whose clinical data were not recorded in detail by their clinicians were excluded; 975 patients had Aβ‐PET and 540 patients had Tau‐PET done, and 220 patients had APOE genotype detection. NPSs were provided by 325 participants across the 12‐item Neuropsychiatric Inventory (NPI) data.

### 2.2 Clinical assessment

The clinical assessment was performed by neurologists specialized in dementia care and included a detailed history taken from the primary caregivers of the patient, a physical examination, cognitive assessments, laboratory tests (including thyroid/liver/kidney function tests, vitamin B12 level, folate level, syphilis serology, and APOE genotype), and neuroimaging (brain MRI/CT and PET‐CT).

Neuropsychological assessments were assessed during the 2 weeks before PET imaging. The Mini‐Mental State Examination‐Chinese version (MMSE), the Montreal Cognitive Assessment (MoCA), and the Clinical Dementia Rating (CDR) scale were used to evaluate global cognitive function and the severity of CI among all of the participants. Scores on the MMSE and MoCA range from 0 (severe impairment) to 30 (no impairment). CDR range from 0.0 (no dementia), 0.5 (MCI), 1.0 (mild), 2.0 (moderate) to 3.0 (severe). NPS were assessed with the 12‐item NPI using the information provided by their caregivers. The composite score of each subscale ranges between 0 (no NPS) and 12 and the total composite score between 0 (no NPS) and 144.

### 2.3 Imaging acquisition

Acquisition procedures for FDG‐PET and Aβ‐PET have been fully described in a previous study.^12^ Briefly, a T1‐weighted sequence was acquired on a 3.0‐T GE Healthcare scanner or a 3.0‐T Siemens Trio, A Tim, MRI scanner. Patients were injected intravenously with 240–333 MBq of ^18^F‐FDG, and a 10‐min static PET scan was obtained 40 min after injection of ^18^F‐FDG. The 3D Aβ PET images were acquired by a Discovery Elite scanner (GE Healthcare) at Beijing Tiantan Hospital or a Siemens Biograph 64 PET/CT scanner at PET center of Huashan Hospital. We analyzed data acquired 40–60 minutes post‐injection for ^11^C‐PIB (at Shanghai Huashan Hospital) and 50 min post‐injection for [^18^F] AV45 (at Beijing Tiantan Hospital or Shanghai Huashan Hospital). Patients were diagnosed as PIB‐positive or AV45‐positive on the basis of both visual interpretations of elevated binding in the neocortex and semiquantitative assessment with standardized uptake value ratio (SUVR) > 1.40 or SUVR >1.11, respectively.

[^18^F]PM‐PBB3 ([^18^F]‐APN‐1607) PET scans were obtained on a Siemens Biograph 64 PET/computed tomography (CT) system (Siemens, Erlangen, Germany) in three‐dimensional (3D) mode at Huashan Hospital. A low‐dose CT transmission scan was performed for attenuation correction. Ninety minutes after intravenous injection of 370 MBq [^18^F]‐APN‐1607, PET imaging was performed with a 20‐min acquisition. Image reconstruction was obtained by a 3‐D ordered‐subset expectation maximization algorithm (4 iterations; 24 subsets; Gaussian filter, 2 mm; zoom, 3). SUVRs were calculated using cerebellar gray matter as reference with SPM12 (http://www.fil.ion.ucl.ac.uk/spm) and cat12 (http://www.neuro.uni‐jena.de/cat/) software implemented in MATLAB 2018b (Mathworks Inc., MA, USA). All PET images were coregistered with T1 images, spatially normalized in the Montreal Neurological Institute template, and then smoothed. The regional SUVR *z* score was defined as: (single patient's SUVR—mean SUVR observed in healthy controls)/SD of SUVR value observed in healthy controls. A regional *z* score ≥ 2 was considered to define positive findings for semiquantitative interpretation at the regional level.^13^ In this study, considering the distribution of positive regions in patients with α‐synucleinopathies and healthy controls, patients with at least two positive regions of interest on ^18^F‐APN‐1607 tau PET imaging (2‐region positivity approach) were diagnosed as [^18^F]‐APN‐1607‐positive.^14^

There was at least a 1‐day interval for each PET scan and a delay of no longer than 14 days between PET and MRI scans. The study participants underwent PET imaging detections after completion of clinical assessments.

### 2.4 APOE genotyping

Genomic DNA was extracted from peripheral blood stored at −80 °C, and the APOE gene was amplified by polymerase chain reaction (PCR), with details in Appendix S1. We determined all genotypes without knowledge of the patient's status.

### 2.5 Diagnostic criteria

Clinical criteria for AD, FTLD, DLB, and VaD were used to establish the initial clinical diagnosis based on the respective diagnostic guidelines. Probable AD dementia was diagnosed according to the criteria of the National Institute on Aging and the Alzheimer Association workgroup.^15^ Consensus criteria for the diagnosis of FTLD were formulated in 1998,^16^ and primary non‐fluent aphasia (PNFA) and semantic dementia (SD) were comprised under the behavioral variant frontotemporal dementia (bvFTD).^17^ The atypical forms including corticobasal syndrome (CBS),^18^ progressive supranuclear palsy (PSP),^19^ and amyotrophic lateral sclerosis (FTLD/ALS)^20^ were diagnosed using respective diagnostic criteria. Patients with probable DLB were diagnosed using the criteria of McKeith in 2017,^4^ and those with VaD were diagnosed according to the NINDS‐AIREN criteria (National Institute of Neurologic Disorders and Stroke/Association International pour la Recherche et al'Enseignement en Neurosciences).^21^

### 2.6 Statistical analysis

**FIGURE 1:**
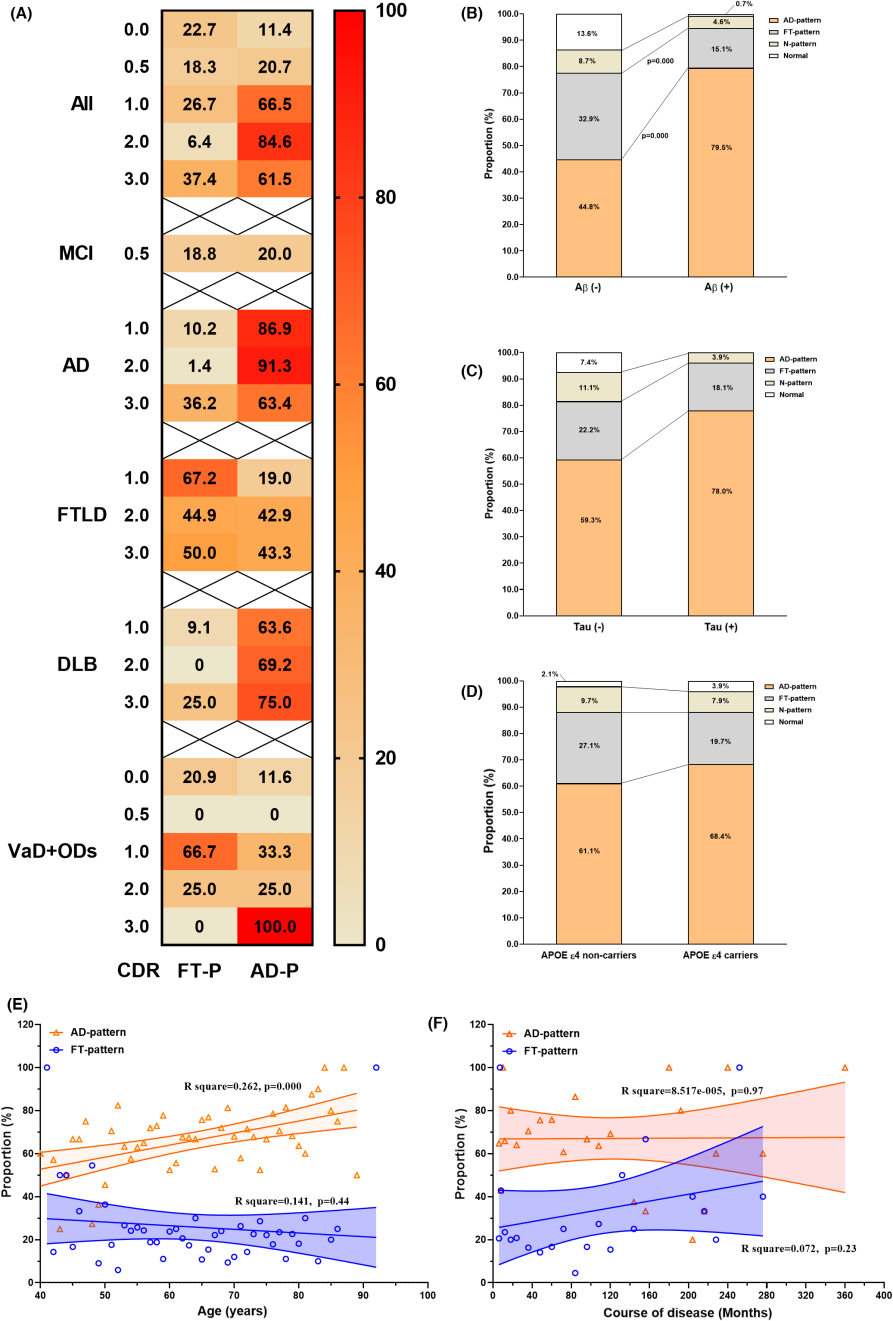
Metabolism pattern in patients with cognitive impairment by demographic and clinical characteristics. The proportions of AD‐P and FT‐P hypometabolism in patients with cognitive impairment by CDR (A), Aβ deposition (B), Tau aggregation (C), or APOE ɛ4 carrier (D) were calculated by descriptive analysis. In (A), the percentage numbers were written in each box, with redder colors representing larger proportions. Chi‐square test was used to compare the proportions of hypometabolism patterns between the two groups, and the significant *p*‐values (after Bonferroni correction) are shown in (B–D). Linear regressions were used to analyze the association between the accumulate frequencies of AD‐P or FT‐P hypometabolism and age (E) or course of disease (F). AD, Alzheimer's disease; AD‐P, AD pattern; APOE ε4, Apolipoprotein ε4; Aβ, amyloid‐β; CDR, clinical dementia rating; DLB, dementia with Lewy bodies; FTLD, frontotemporal lobar degeneration; FT‐P, frontotemporal area hypometabolism; MCI, mild cognitive impairment; N‐pattern, non‐specific pattern; ODs, other diagnosis; VaD, vascular dementia.

The Skewness–Kurtosis test was used to check the normal distribution of the data. Since the age, education years, course of disease, the scores of MMSE and MoCA did not satisfy the normal distribution, the data were described as the medians (interquartile range, IQR). The qualitative variables were expressed as frequency, and the chi‐squared test was used to compare the two independent groups (Figure 1B–D) for qualitative variables. Linear regressions were run to analyze the associations between accumulated frequencies of hypometabolic patterns and age at PET‐CT performed (Figure 1E) and the course of disease (Figure 1F). The correlation between clinical features and metabolism pattern was evaluated by chi‐squared tests and described by Pearson contingency coefficient for two qualitative variables (such as the correlations between AD‐P/FT‐P and Aβ deposition/Tau aggregation/APOE ε4 carrier/12 items of NPI in Table 3), or Spearman's correlation between qualitative variables and qualitative variables (such as the correlations between AD‐P/FT‐P and scores of MMSE, MoCA, and NPI in Table 3). Multivariable logistic regression was used to produce individual predicted probability using the cross‐validated method of the leave‐one‐out principle, which drops the data of one subject and re‐estimates the parameters. The cross‐validated predicted probabilities were used to assess discriminatory performance of hypometabolism of AD‐P or FT‐P, amyloid deposition, and Tau aggregation in AD/FTLD with other dementia subtypes by areas under the receiver operating characteristic (ROC) curve (AUC).

For the statistical analyses, the IBM SPSS for Windows (version 25.0; IBM Corporation, Armonk, NY, USA) was used. *p* Values of <0.05 are considered significant at the two‐tailed α level. Data were analyzed from January to May 2022.

## 3 RESULTS

In total, 1037 patients with CI (475 women [45.8%] and 562 men [54.2%]; mean [SD] age, 64.5 [9.8] years) were recruited, of whom 80 had MCI, 741 had clinical AD, 137 had FTLD, 28 had DLB, 5 had VaD, and 46 had other diagnoses (Table 1). Of the 975 (94.0%) CI patients with Aβ‐PET performed by ^11^C‐PIB PET or ^18^F‐AV45 PET, 70.7% (689/975) of patients were Aβ‐positive. AD and DLB had higher proportions of Aβ deposition, with 86.1% (638/689) and 84.6% (11/13), respectively. Of the 540 (52.1%) CI patients with [^18^F]PM‐PBB3 ([^18^F]‐APN‐1607) PET, 95.0% (513/540) of patients were Tau‐positive, and over 90.0% of patients with VaD (3/3, 100.0%), FTLD (135/137, 98.5%), clinical AD (359/370, 97.0%), or DLB (12/13, 92.3%) were Tau‐positive, respectively. Clinical AD patients comprised 49.5% (54/109) of APOE ε4 carriers. The NPS of all participants are displayed in Table S1.

**TABLE 1:** Basic demographic characteristics.

Characteristics^a^	All (*n* = 1037)	MCI (*n* = 80)	AD (*n* = 741)	FTLD (*n* = 137)	DLB (n = 28)	VaD (n = 5)	ODs (n = 46)
Sex							
Men	562 (54.2)	43 (53.8)	415 (56.0)	64 (46.7)	14 (50.0)	3 (60.0)	23 (50.0)
Women	475 (45.8)	37 (46.3)	326 (44.0)	73 (53.3)	14 (50.0)	2 (40.0)	23 (50.0)
Age at PET‐CT performed, mean (SD), y	64.0 (57.0, 72.0)	61.0 (55.3, 68.0)	65.0 (58.0, 73.0)	62.0 (55.0, 68.0)	67.0 (60.3, 73.0)	74.0 (54.0, 75.0)	60.0 (52.3, 68.3)
Age of onset, mean (SD), y	62.0 (55.0, 67.0)	59.5 (53.0, 62.0)	62.0 (55.0, 68.0)	60.0 (52.5, 65.0)	62.5 (58.0, 70.0)	72.0 (52.5, 72.5)	58.0 (49.0, 64.3)
Course of disease, mean (SD), months	24.0 (12.0, 48.0)	24.0 (12.0, 36.0)	24.0 (24.0, 48.0)	24.0 (12.0, 24.0)	24.0 (18.0, 45.0)	24.0 (18.0, 30.0)	24.0 (12.0, 36.0)
Education level	11.0 (10.0, 12.0)	11.0 (11.0, 12.0)	10.0 (10.0, 12.0)	12.0 (9.0, 12.0)	9.0 (6.0, 12.0)	12.0 (7.5, 12.0)	12.0 (12.0, 12.0)
Illiteracy	6 (0.6)	0 (0.0)	2 (0.3)	4 (2.8)	0 (0.0)	0 (0.0)	0 (0.0)
Primary school	54 (5.2)	1 (1.3)	25 (3.4)	15 (10.9)	9 (32.1)	1 (20.0)	3 (6.5)
Junior high school and above	977 (94.2)	79 (98.7)	714 (96.3)	118 (86.3)	19 (67.9)	4 (80.0)	43 (93.5)
MMSE (*n* = 446), mean (SD)	20.0 (14.0, 24.0)	26.0 (23.8, 28.0)	17.0 (12.0, 22.0)	20.0 (13.0, 24.0)	18.0 (13.5, 22.3)	15.0 (15.0, 15.0)	28.0 (26.0, 29.8)
MOCA (*n* = 387), mean (SD)	12.0 (7.0, 18.0)	21.5 (18.0, 25.0)	11.0 (7.0, 15.0)	11.0 (7.0, 17.8)	11.0 (6.5, 15.3)	Median = 11.5	26.0 (20.5, 28.0)
CDR	2.0 (1.0, 3.0)	0.5 (0.5, 0.5)	2.0 (2.0, 3.0)	2.0 (1.0, 2.0)	2.0 (1.0, 2.0)	2.0 (1.5, 2.0)	0.0 (0.0, 0.0)
0.0	44 (4.2)	0 (0.0)	0 (0.0)	0 (0.0)	0 (0.0)	0 (0.0)	43 (93.5)
0.5	82 (7.9)	80 (100.0)	0 (0.0)	0 (0.0)	0 (0.0)	0 (0.0)	0 (0.0)
1.0	206 (19.9)	0 (0.0)	137 (18.5)	58 (42.3)	11 (39.3)	1 (20.0)	2 (4.3)
2.0	435 (41.9)	0 (0.0)	369 (49.8)	49 (35.8)	13 (46.4)	4 (80.0)	0 (0.0)
3.0	270 (26.0)	0 (0.0)	235 (31.7)	30 (21.9)	4 (14.3)	0 (0.0)	1 (2.2)
Aβ deposition (*n* = 975)							
Negative	286 (29.3)	51 (73.9)	103 (13.9)	117 (85.4)	2 (15.4)	3 (60.0)	10 (100.0)
Positive	689 (70.7)	18 (26.1)	638 (86.1)	20 (14.6)	11 (84.6)	2 (40.0)	0 (0.0)
Tau aggregation (*n* = 540)							
Negative	27 (5.0)	10 (83.3)	11 (3.0)	2 (1.5)	1 (7.7)	0 (0.0)	3 (60.0)
Positive	513 (95.0)	2 (16.7)	359 (97.0)	135 (98.5)	12 (92.3)	3 (100.0)	2 (40.0)
APOE ε4 carrier (*n* = 220)							
No	144 (65.5)	7 (58.3)	55 (50.5)	56 (81.2)	14 (93.3)	2 (100.0)	10 (76.9)
Yes	76 (34.5)	5 (41.7)	54 (49.5)	13 (18.8)	1 (6.7)	0 (0.0)	3 (23.1)

Abbreviations: AD, Alzheimer's disease; APOE ε4, Apolipoprotein ε4; Aβ, Amyloid‐β; CDR, the clinical dementia rating; DLB, dementia with Lewy bodies; FTLD, frontotemporal lobar degeneration; MCI, mild cognitive impairment; MMSE, the Mini‐Mental State Examination; MoCA, Montreal Cognitive Assessment Scale; ODs, other diagnosis; PET CT, Positron Emission Tomography‐Computed Tomography; SD, standard deviation; VaD, vascular dementia.

^a^ Unless otherwise indicated, data are expressed as number (%) of participants. Owing to missing data, the sample total may not equal the total number in the column headings, so we make supplementary number in the first column.

66.8% (693/1037) of patients showed AD‐P hypometabolism predominantly in the posterior regions, including the posterior temporoparietal association cortex and posterior cingulate cortex, with or without frontal lobe involvement, and 20.2% (209/1037) of patients showed FT‐P hypometabolism predominantly in the anterior regions, including the frontal and anterior temporal areas, anterior cingulate gyrus, and insula (Table 2). A total of 81.6% (605/741) AD patients and 67.9% (19/28) DLB patients showed AD‐P hypometabolism. Of the 137 FTLD patients, 55.5% (76/137) showed FT‐P hypometabolism, and 32.8% (45/137) showed AD‐P hypometabolism. After classification of the diagnosis of FTLD, 62.5% (10/16) of patients with CBD showed more AD‐P hypometabolism, while patients with bvFTD (62.1%, 36/58), SD (70.0%, 7/10), PNFA (66.7%, 6/9), FTLD/ALS (60.0%, 3/5), and PSP (56.8%, 21/37) showed mostly FT‐P hypometabolism. Of the two patients with PPA, one showed AD‐P hypometabolism, and the other showed FT‐P hypometabolism.

**TABLE 2:** Brain hypometabolism pattern in all participants.

Hypometabolism pattern^a^	All (*n* = 1037)	MCI (*n* = 80)	AD (*n* = 741)	FTLD								DLB (*n* = 28)	VaD (*n* = 5)	ODs (*n* = 46)
				All (*n* = 137)	bvFTD (*n* = 58)	SD (*n* = 10)	PNFA (*n* = 9)	PPA (*n* = 2)	CBD (*n* = 16)	FTLD/ALS (*n* = 5)	PSP (*n* = 37)			
AD‐P	693 (66.8)	16 (20.0)	605 (81.6)	45 (32.8)	19 (32.8)	2 (20.0)	3 (33.3)	1 (50.0)	10 (62.5)	1 (20.0)	9 (24.3)	19 (67.9)	2 (40.0)	6 (13.0)
FT‐P	209 (20.2)	15 (18.8)	104 (14.0)	76 (55.5)	36 (62.1)	7 (70.0)	6 (66.7)	1 (50.0)	2 (12.5)	3 (60.0)	21 (56.8)	2 (7.1)	1 (20.0)	11 (23.9)
N‐P	85 (8.2)	9 (11.3)	28 (3.8)	16 (11.7)	3 (5.2)	1 (10.0)	0 (0.0)	0 (0.0)	4 (25.0)	1 (20.0)	7 (18.9)	6 (21.4)	2 (40.0)	24 (52.2)
Normal	50 (4.8)	40 (50.0)	4 (0.5)	0 (0.0)	0 (0.0)	0 (0.0)	0 (0.0)	0 (0.0)	0 (0.0)	0 (0.0)	0 (0.0)	1 (3.6)	0 (0.0)	5 (10.9)

Abbreviations: AD, Alzheimer's disease; AD‐P, AD pattern hypometabolism; bvFTD, behavioral variant frontotemporal dementia; CBD, cortical baseal degeneration; DLB, dementia with Lewy bodies; FTLD, frontotemporal lobar degeneration; FTLD/ALS, frontotemporal lobar degeneration and amyotrophic lateral sclerosis; FT‐P, frontotemporal lobe‐pattern hypometabolism; MCI, mild cognitive impairment; N‐P, non‐specific hypometabolism; ODs, other diagnosis; PNFA, progressive non‐fluent aphasia; PPA, Primary progressive aphasia; PSP, progressive supranuclear palsy; SD, semantic dementia; VaD, vascular dementia.

^a^ Data are expressed as number (%) of participants.

Figure 1 and Table S2 show that patients with Aβ deposition, Tau aggregation, or APOE ε4 allele had higher probabilities of AD‐P hypometabolism. In addition, the proportion of AD‐P hypometabolism in all CI patients was significantly positively related to age (*R* ^2^ = 0.262, *p* = 0.000) but not the course of the disease.

The proportions of AD‐P and FT‐P hypometabolism were associated with Aβ deposition, Tau aggregation, total scores of MMSE and MoCA, and the presence of apathy and appetite and eating abnormalities in the 325 patients with NPI evaluation. The proportion of AD‐P hypometabolism was associated with MMSE and MoCA total scores in MCI and clinical AD. In addition, the presence of APOE ε4 allele and hallucinations assessed by NPI were associated with the proportion of AD‐P hypometabolism. Furthermore, the proportion of FT‐P hypometabolism was associated with the presence of hallucinations (*R* = 0.171, *p* = 0.04), anxiety (*R* = 0.182, *p* = 0.03), and appetite and eating abnormalities (*R* = 0.200, *p* = 0.01) in AD (Table 3).

**TABLE 3:** Relationship between clinical features and metabolism pattern in all participants.

Variables	Coefficients & *p*‐values	All (*n* = 325)		MCI (*n* = 32)		AD (*n* = 148)		FTLD (*n* = 93)		DLB (*n* = 23)		VaD+ ODs (*n* = 28)	
		AD‐P	FT‐P	AD‐P	FT‐P	AD‐P	FT‐P	AD‐P	FT‐P	AD‐P	FT‐P	AD‐P	FT‐P
Aβ deposition	*R*	**0.343**	**−0.201**	**0.642**	−0.033	−0.059	0.016	**0.195**	−0.129	**0.778**	0.123	0.294	0.207
	*p*‐value	**0.000**	**0.000**	**0.000**	0.79	0.11	0.66	**0.02**	0.13	**0.002**	0.69	0.29	0.46
Tau aggregation	*R*	0.097	−0.023	0.000	0.258	0.038	−0.045	0.085	−0.109	0.433	0.083	0.067	0.258
	*p*‐value	0.024	0.59	1.00	0.42	0.47	0.39	0.32	0.21	0.14	0.79	0.88	0.54
APOE ε4 carrier	*R*	0.072	−0.081	0.371	−0.255	**−0.223**	**0.214**	−0.026	0.016	0.134	−0.071	−0.250	−0.200
	*p*‐value	0.29	0.23	0.24	0.42	**0.02**	**0.03**	0.83	0.90	0.64	0.80	0.37	0.47
MMSE score	*R*	**−0.354**	**0.216**	**−0.488**	0.231	**−0.202**	**0.165**	−0.154	0.118	−0.361	0.339	−0.358	0.317
	*p*‐value	**0.000**	**0.000**	**0.001**	0.14	**0.001**	**0.007**	0.14	0.26	0.12	0.14	0.10	0.15
MoCA score	*R*	**−0.310**	**0.146**	**−0.510**	0.304	**−0.172**	0.125	−0.126	0.124	−0.451	0.379	−0.299	0.187
	*p*‐value	**0.000**	**0.004**	**0.001**	0.07	**0.01**	0.06	0.24	0.25	0.06	0.12	0.18	0.40
NPI score	*R*	0.047	0.042	−0.283	**0.654**	−0.038	0.102	0.091	−0.07	0.246	−0.108	−0.062	−0.169
	*p*‐value	0.40	0.45	0.12	**0.000**	0.65	0.22	0.38	0.50	0.26	0.63	0.76	0.39
Delusions	*R*	0.081	0.011	–	–	0.037	0.017	0.113	−0.054	−0.359	−0.066	−0.064	0.262
	*p*‐value	0.13	0.84	–	–	0.65	0.84	0.26	0.59	0.09	0.77	0.69	0.09
Hallucinations	*R*	0.084	−0.051	–	–	**−0.178**	**0.171**	0.088	−0.128	0.190	−0.099	−0.064	0.262
	*p*‐value	0.13	0.34	–	–	**0.03**	**0.04**	0.37	0.20	0.39	0.65	0.69	0.09
Agitation	*R*	−0.003	0.068	–	–	−0.128	0.147	−0.056	0.095	−0.358	−0.120	–	–
	*p*‐value	0.96	0.20	–	–	0.12	0.07	0.58	0.34	0.09	0.59	–	–
Depression	*R*	0.000	0.066	−0.131	0.147	−0.045	0.134	0.078	0.005	−0.324	0.168	0.179	−0.128
	*p*‐value	1.00	0.22	0.47	0.42	0.58	0.10	0.43	0.96	0.13	0.44	0.26	0.42
Anxiety	*R*	0.06	−0.004	−0.271	**0.528**	−0.147	**0.182**	**0.236**	−0.140	0.172	0.013	0.060	−0.219
	*p*‐value	0.26	0.94	0.13	**0.002**	0.07	**0.03**	**0.02**	0.16	0.43	0.95	0.71	0.16
Euphoria	*R*	0.032	−0.010	–	–	−0.094	−0.042	0.148	−0.076	–	–	**0.383**	−0.093
	*p*‐value	0.55	0.85	–	–	0.25	0.61	0.13	0.44	–	–	**0.01**	0.56
Apathy	*R*	**0.121**	0.012	–	–	−0.058	0.086	0.159	−0.096	0.398	0.225	−0.113	0.255
	*p*‐value	**0.02**	0.82	–	–	0.48	0.29	0.11	0.33	0.06	0.30	0.48	0.10
Disinhibition	*R*	0.026	0.021	–	–	−0.046	−0.055	0.174	−0.110	0.127	−0.066	**0.383**	−0.093
	*p*‐value	0.63	0.70	–	–	0.58	0.51	0.08	0.26	0.57	0.77	**0.01**	0.56
Irritability	*R*	0.059	0.023	0.039	−0.094	−0.072	0.037	−0.020	0.092	0.011	−0.142	**0.383**	−0.093
	*p*‐value	0.27	0.67	0.83	0.60	0.38	0.65	0.84	0.35	0.96	0.52	**0.01**	0.56
Aberrant motor behavior	*R*	0.012	**0.115**	–	–	−0.137	0.156	0.039	0.053	0.073	0.211	–	–
	*p*‐value	0.82	**0.03**	–	–	0.10	0.06	0.70	0.60	0.74	0.33	–	–
Night‐time behavior disturbances	*R*	−0.007	0.012	−0.058	**0.528**	−0.014	0.079	0.092	−0.074	0.321	−0.271	0.021	−0.165
	*p*‐value	0.90	0.82	0.75	**0.002**	0.87	0.34	0.35	0.45	0.14	0.21	0.90	0.30
Appetite and eating abnormalities	*R*	−0.049	**0.147**	–	–	−0.132	**0.200**	0.008	0.095	−0.359	−0.066	–	–
	*p*‐value	0.36	**0.006**	–	–	0.11	**0.01**	0.94	0.34	0.09	0.77	–	–

*Note*: It demonstrated the relationship between AD‐P/FT‐P hypometabolism and Aβ deposition, Tau deposition, APOE ɛ4 carriers, scores of MMSE, MoCA, NPI, and its items in all the participants with cognitive impairment. “*R*” represented the coefficients calculated by chi‐squared tests or Spearman's correlation. All bold values in this table represented having significant difference.

Abbreviations: AD, Alzheimer's disease; AD‐P, AD pattern hypometabolism; APOE ε4, Apolipoprotein ε4; Aβ, amyloid‐β; DLB, dementia with Lewy bodies; FTLD, frontotemporal lobar degeneration; FT‐P, frontotemporal lobe‐pattern hypometabolism; MCI, mild cognitive impairment; MMSE, the Mini‐Mental State Examination; MoCA, Montreal Cognitive Assessment Scale; NPI, Neuropsychiatric Inventory; ODs, other diagnosis; VaD, vascular dementia.

**FIGURE 2:**
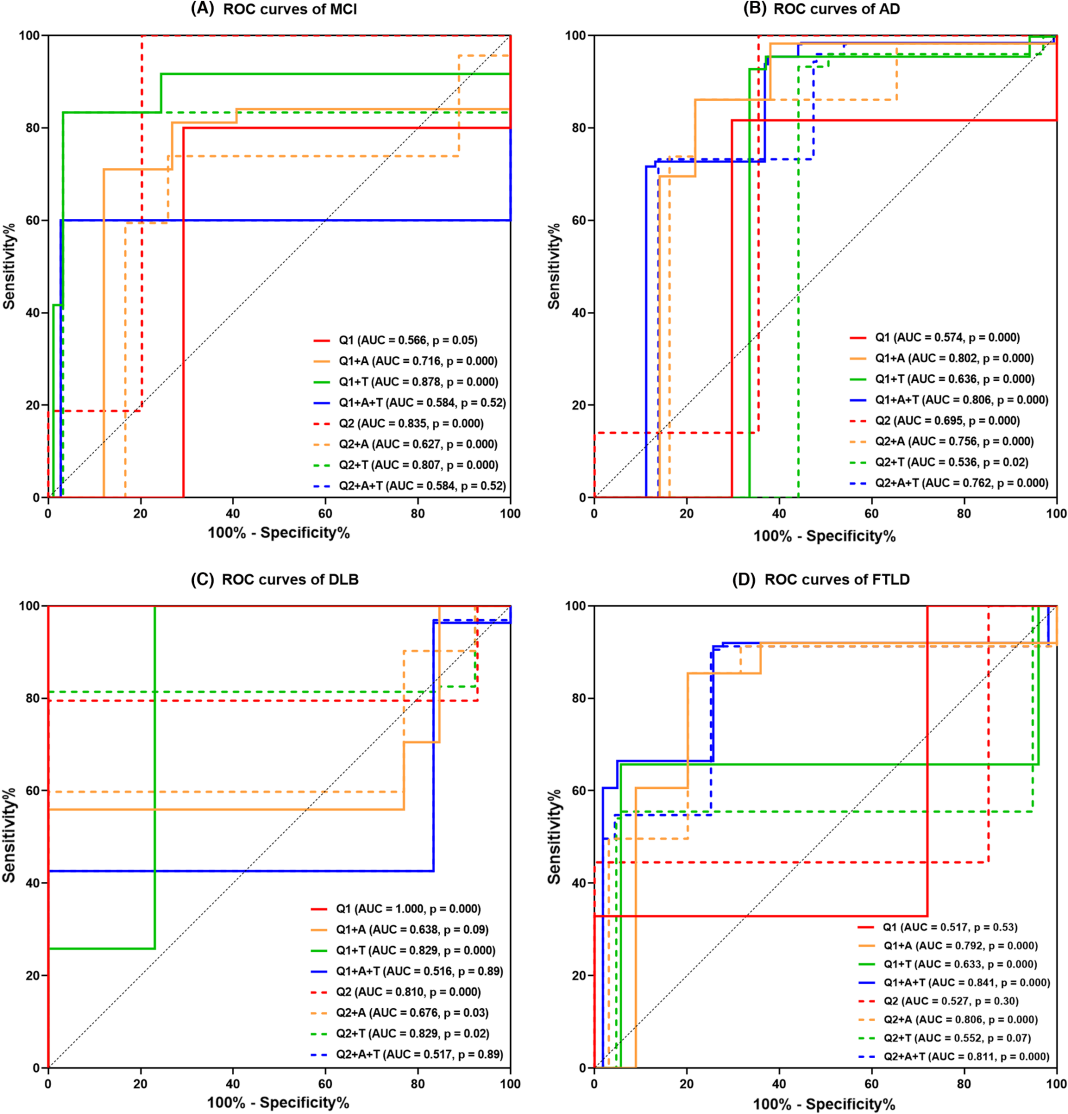
The potential discriminatory performance of hypometabolism patterns and biomarkers in cognitive impairment. The receiver operating characteristic curves of MCI (A), AD (B), DLB (C), and FTLD (D) were shown. Dashed gray lines represent the baseline curve. Q1 means AD pattern hypometabolism; Q2 means frontal and anterior temporal area hypometabolism; “A” represents amyloid‐β deposition and “T” represents Tau aggregation. AD, Alzheimer's disease; AUC, area under the curve; DLB, dementia with Lewy bodies; FTLD, frontotemporal lobar degeneration; MCI, mild cognitive impairment.

The ROC curves in Figure 2 revealed that the application of AD‐P or FT‐P hypometabolism, Aβ‐PET, and [^18^F]PM‐PBB3 ([^18^F]‐APN‐1607) PET could detect the discriminatory performance of AD [the max AUCs = 0.806 (95%CI: 0.754–0.857)], FTLD [the max AUCs = 0.841 (95%CI: 0.794–0.889)] from other subtypes of CI.

## 4 DISCUSSION

In this multicenter neuroimaging cohort study among patients with CI, the majority of AD, DLB, and CBD patients demonstrated AD‐P hypometabolism, while FTLD and its main subtypes, including bvFTD, SD, PNFA, FTLD/ALS, and PSP, showed predominantly FT‐P hypometabolism, whereas no specific hypometabolic patterns were found in MCI. The combination of metabolic pattern and biomarkers can effectively distinguish AD/FTLD from other types of dementia. In addition, the higher proportion of AD‐P hypometabolism was associated with old age, Aβ deposition, Tau aggregation, and worse cognition, although not with APOE ε4 allele or the course of disease. Furthermore, a higher proportion of FT‐P hypometabolism was related to the presence of hallucinations, anxiety, and appetite and eating abnormalities in AD.

### 4.1 Interpretation of results

In the present study, over half of patients with clinical AD and DLB showed AD‐P hypometabolism, which was consistent with previous neuroimaging research.^22^, ^23^ In addition, hypometabolism in the lateral occipital cortex and the “cingulate island sign” specifically predicted DLB.^4^ Hypometabolism depicted by FDG‐PET reflects reduced neuronal activity in general, and recent criteria have supported its use in staging the disease and labeled FDG‐PET as a downstream biomarker of degeneration.^24^ However, we did not find an association between AD‐P hypometabolism and the course of disease. As previous work has reported,^25^ age‐related hypometabolism in AD‐P is mainly in the anterior cingulate and anterior temporal lobe. Hypometabolism in the posterior cingulate, inferior parietal lobe, and precuneus is a predictor of cognitive decline from MCI to AD. As previously reported,^26^ a significant decreased metabolism in the precuneus was detected in patients with autosomal dominant AD (one of PSEN 1, PSEN2, or APP genes mutation) 10 years before progressive cognitive decline onset, whereas hypometabolism was comparable to or several years after progressive cognitive decline for those without mutations. This may explain why we did not find significantly specific hypometabolism patterns for patients with non‐mutation MCI after progressive cognitive decline onset. Moreover, the Braak stages of Aβ and Tau deposition often came with glucose metabolic imaging in AD.^27^ PIB‐PET demonstrated a negative association between brain reserve and AD pathological burden, showing as a significantly higher accumulation of PIB and lower glucose metabolic rates in the parietotemporal cortical regions, medial frontal, anterior, and posterior cingulate gyri.^27^, ^28^ A higher 18F‐FDG PET cingulate island sign ratio was associated with lower Braak tangle stage at autopsy in DLB.^29^ All these findings indicated the glucose hypometabolism associated with the extent of Alzheimer's pathology. Even though the proportions of AD‐P and FT‐P hypometabolism were found to be associated with Aβ deposition and Tau aggregation in this study, it cannot fully represent the accurate relationship between the hypometabolic pattern and AD pathological burden due to the high proportions of Aβ‐positive and Tau‐positive in this population, and more longitudinal studies are still needed to confirm it.

Patients with FTLD predominantly demonstrated extensive cortical hypometabolism in the frontal and anterior temporal areas, cingulate gyri, uncus, insula, and subcortical areas, including the basal ganglia (putamen and globus pallidus) and medial thalamic regions. Our results found that 55.5% FTLD patients performed FT‐P hypometabolism, and 32.8% showed AD‐P hypometabolism. The proportion of frontal hypometabolism was lower than literature^30^, ^31^ possibly due to the FT‐P and AD‐P classification in this study. In some patients with severe FTLD, in addition to hypometabolism in the frontal and temporal lobes, posterior temporoparietal association cortex and posterior cingulate cortex will also be involved. Therefore, it was classified as “AD‐P hypometabolism” according to the grouping, leading to the inconsistence with previous studies. And in the language variant FTLD (SD, PNFA, and PPA in our study), cerebral glucose metabolism was reduced exclusively in the temporal lobes, with or without frontal hypometabolism. The literature shows that the metabolic patterns in CBD and PSP can reflect the area that is affected by the pathology of each disease. Of the 16 patients with CBD, 10 showed AD‐P hypometabolism, likely reflecting existing AD pathology, which is consistent with our previous report.^12^ In addition to midbrain hypometabolism, 21 of 37 patients with PSP showed FT‐P hypometabolism, especially in the frontal lobe. Among the five patients with FTLD/ALS, one showed AD‐P hypometabolism, three showed FT‐P hypometabolism, and one showed non‐specific hypometabolism. Similarly, Canosa et al. demonstrated a large cluster of patients with relative hypometabolism in the frontal lobe in PSP, and they pointed to frontal hypometabolism as a marker to reflect the clinical and neuropathological continuum ranging from pure ALS to FTLD/ALS.^32^

Regional glucose metabolism in FDG‐PET is reported in association with NPS. For instance, frontal region hypometabolism is observed in preclinical AD, MCI, and AD patients with depression.^33^ The presence of apathy is associated with decreased metabolism in the posterior cingulate cortex, frontal, temporal, and cerebellar areas,^34^, ^35^ and the amygdala circuit is related to anxiety.^36^ The reduced occipital metabolism frequently seen in DLB is associated with frequency and severity of visual hallucinations.^37^ Of our 325 patients with both NPI and FDG‐PET, FT‐P hypometabolism was significantly related to the presence of anxiety and night‐time behavior disturbances in MCI as well as the presence of hallucinations, anxiety, and appetite and eating abnormalities in AD. Previous FDG‐PET studies^38^, ^39^ indicated that higher anxiety was associated with lower metabolism in the medial temporal lobe, particularly the bilateral entorhinal cortex, bilateral anterior parahippocampal gyrus, and left anterior superior temporal gyrus in AD and MCI. Several studies have shown an association of hallucination with hypometabolism right ventral and dorsolateral prefrontal area^40^, ^41^ and right temporal.^42^ Other neuroimaging studies found appetite and eating abnormalities were associated with hypometabolism in the orbitofrontal cortex,^43^, ^44^ or the atrophy of the medial temporal cortex.^45^ However, we cannot demonstrate the significant associations between FT‐P hypometabolism with other NPS like previous literature^9^, ^10^, ^11^, ^36^, ^46^ since the differences in target population or sample‐size.

These findings have potential clinical and programmatic relevance for standardizing early diagnosis of dementia, and improving the accuracy of differential diagnosis. The significant differences in hypometabolic patterns across CI types makes FDG‐PET a helpful parameter in the differential diagnosis of CI, while the independent application of AD‐P and FT‐P hypometabolism plays a minor role in the diagnosis of MCI and DLB.

### 4.2 Limitations

We present a multicenter cohort study with comprehensive neurological assessment and molecular imaging biomarkers in a large group of CI patients. First, a little part of the target participants had “clinical diagnosis” due to the absence of blood and CSF biomarkers, it might weaken the strength of our finding. Moreover, the main limitation in this study is the qualitative interpretation of the FDG‐PET images with AD‐P or FT‐P and the lack of amyloid and Tau deposition pattern. The lack of detailed descriptions of brain regions limited us from doing more analysis, likely a detailed evaluation of the relationship among NPS, hypometabolic areas, Aβ, and Tau deposition patterns.

## 5 CONCLUSION

These results suggest that brain hypometabolic patterns are (i) significantly different across CI types, (ii) associated with old age, Aβ deposition, Tau aggregation, and worse cognition, and (iii) related to the presence of hallucination, anxiety, and appetite and eating abnormalities in AD. In addition, the combined application of AD‐P or FT‐P hypometabolism and biomarkers can effectively distinguish AD/FTLD from other types of dementia. Furthermore, specific hypometabolic patterns are associated with NPS and beneficial for the early identification and management of NPS in patients with CI.

## AUTHOR CONTRIBUTIONS

Dr. YJ had full access to all of the data in the study and take responsibility for the integrity of the data and the accuracy of the data analysis. Concept and design: YJ and YG. Acquisition, analysis, or interpretation of data: All authors. Drafting of the manuscript: JG. Critical revision of the manuscript for important intellectual content: ZS, LA, and NZ. Statistical analysis: YC, JG, and SL. Obtained funding: YJ and ZS. Administrative, technical, or material support: CZ, XZ, LC, and RC.

## CONFLICT OF INTEREST STATEMENT

The authors declare that they have no competing interests.

## Supporting information


Appendix S1

## Data Availability

The data that support the findings of this study are available from the corresponding author upon reasonable request.
