# Supplementary material for: Analysis of positron emission tomography hypometabolic patterns and neuropsychiatric symptoms in patients with dementia syndromes
Source: CNS Neurosci Ther. 2023 Mar 16;29(8):2193–205. doi: 10.1111/cns.14169 (PMC10352896; doi:10.1111/cns.14169)
Supplement: Supplementary file 1 — Appendix S1 [file CNS-29-2193-s001.docx]

**eAppendix 1**

**Details of APOE genotyping**

Genomic DNA was extracted from peripheral blood stored at -80 ℃, and the APOE gene was amplified by polymerase chain reaction (PCR). The PCR primers were: 5′-AGGAACAACTGACCCCGGT-3′ (upstream) and 5′-TGCTCCTTCACCTCGTCCA-3′ (downstream). Each amplification reaction contained 1 µl DNA, 1 ul primers, 1 µl of 10% dNTP, 5 µl Taq Buffer, 5 µl 25 mM MgCl_2_, 0.5 µl Taq DNA polymerase (5 U/µl), and 35.5 µl double-distilled water. The thermal reactor was programmed as follows: initial denaturation at 94°C for 3 min, 35 cycles at 94°C for 30 s, annealing at 57°C for 35 s, extension at 72°C for 40s, and final extension at 72°C for 10 min. The amplification product (50 μl) was purified by a product purification kit (SK 1141). The purified product was sequenced by ABI 3730xl DNA analyzer. We determined all genotypes without knowledge of the patient status.

**Supplement table 1** NPI features of participants (n=325)

| **NPI items (yes, n, %)** | **MCI**  **(n=32)** | **AD**  **(n=148)** | **FTLD (n=94)** | | | | | | | | **DLB**  **(n=23)** | **VaD**  **(n=2)** | **ODs**  **(n=26)** |
| --- | --- | --- | --- | --- | --- | --- | --- | --- | --- | --- | --- | --- | --- |
|  |  |  | **All**  **(n=94)** | **bvFTD**  **(n=46)** | **SD**  **(n=5)** | **PNFA**  **(n=8)** | **PPA**  **(n=2)** | **CBD**  **(n=14)** | **FTLD-ALS**  **(n=5)** | **PSP**  **(n=14)** |  |  |  |
| **Delusions** | 0 (0.0) | 22 (14.9) | 14 (14.9) | 7 (15.2) | 1 (20.0) | 0 (0.0) | 0 (0.0) | 2 (14.3) | 1 (20.0) | 3 (21.4) | 1 (4.3) | 0 (0.0) | 1 (3.8) |
| **Hallucinations** | 0 (0.0) | 13 (8.8) | 4 (4.3) | 1 (2.2) | 0 (0.0) | 0 (0.0) | 0 (0.0) | 1 (7.1) | 0 (0.0) | 2 (14.3) | 15 (65.2) | 0 (0.0) | 1 (3.8) |
| **Agitation** | 0 (0.0) | 23 (15.5) | 13 (13.8) | 10 (21.7) | 0 (0.0) | 2 (25.0) | 0 (0.0) | 0 (0.0) | 1 (20.0) | 0 (0.0) | 3 (13.0) | 0 (0.0) | 0 (0.0) |
| **Depression** | 4 (12.5) | 34 (23.0) | 27 (28.7) | 7 (15.2) | 2 (40.0) | 3 (35.7) | 1 (50.0) | 8 (57.1) | 2 (40.0) | 4 (28.6) | 6 (26.1) | 1 (50.0) | 6 (23.1) |
| **Anxiety** | 3 (9.4) | 27 (18.2) | 18 (19.1) | 5 (10.9) | 1 (20.0) | 1 (12.5) | 1 (50.0) | 5 (35.7) | 1 (20.0) | 4 (28.6) | 11 (47.8) | 0 (0.0) | 5 (19.2) |
| **Euphoria** | 0 (0.0) | 3 (2.0) | 5 (5.3) | 3 (6.5) | 0 (0.0) | 0 (0.0) | 0 (0.0) | 0 (0.0) | 0 (0.0) | 2 (14.3) | 0 (0.0) | 1 (50.0) | 0 (0.0) |
| **Apathy** | 0 (0.0) | 32 (21.6) | 23 (24.5) | 7 (15.2) | 1 (20.0) | 2 (25.0) | 0 (0.0) | 7 (50.0) | 1 (20.0) | 5 (35.7) | 15 (65.2) | 1 (50.0) | 2 (7.7) |
| **Disinhibition** | 0 (0.0) | 5 (3.4) | 14 (14.9) | 8 (17.4) | 0 (0.0) | 4 (50.0) | 0 (0.0) | 1 (7.1) | 1 (20.0) | 0 (0.0) | 1 (4.3) | 1(50.0) | 0 (0.0) |
| **Irritability** | 2 (6.3) | 30 (20.3) | 18 (19.1) | 10 (21.7) | 2 (40.0) | 0 (0.0) | 0 (0.0) | 3 (21.4) | 1 (20.0) | 1 (7.1) | 4 (17.4) | 1 (50.0) | 0 (0.0) |
| **Aberrant motor**  **behavior** | 0 (0.0) | 22 (14.9) | 21 (22.3) | 14 (30.4) | 0 (0.0) | 1 (12.5) | 0 (0.0) | 2 (14.3) | 1 (20.0) | 3 (21.4) | 5 (21.7) | 0 (0.0) | 0 (0.0) |
| **Night-time behavior**  **disturbances** | 3 (9.4) | 23 (15.5) | 23 (24.5) | 10 (21.7) | 2 (40.0) | 3 (37.5) | 0 (0.0) | 5 (35.7) | 1 (20.0) | 2 (14.3) | 10 (43.5) | 1 (50.0) | 8 (30.8) |
| **Appetite and eating**  **abnormalities** | 0 (0.0) | 11 (7.4) | 13 (13.8) | 9 (19.6) | 0 (0.0) | 1 (12.5) | 0 (0.0) | 1 (7.1) | 0 (0.0) | 2 (14.3) | 1 (4.3) | 0 (0.0) | 0 (0.0) |

Abbreviations: NPI, Neuropsychiatric Inventory; MCI, mild cognitive impairment; AD, Alzheimer’s disease; FTLD, frontotemporal lobar degeneration; DLB, dementia with Lewy bodies; VaD, vascular dementia; ODs, other diagnosis; bvFTD, behavioral variant frontotemporal dementia; SD, semantic dementia; PNFA, progressive non-fluent aphasia; PPA, Primary progressive aphasia; CBD, cortical baseal degeneration; FTLD/ALS, frontotemporal lobar degeneration and amyotrophic lateral sclerosis; PSP, progressive supranuclear palsy.

**Supplement table 2** Brain hypometabolic patterns across Aβ deposition, Tau aggregation, and APOE ε4 allele

| **Patterns**  **Variables** | | **MCI** | **AD** | **FTLD** | | | | | | | | **DLB** | **VaD+ODs** |
| --- | --- | --- | --- | --- | --- | --- | --- | --- | --- | --- | --- | --- | --- |
|  |  |  |  | **All** | **bvFTD** | **SD** | **PNFA** | **PPA** | **CBD** | **FTLD/ALS** | **PSP** |  |  |
| Aβ  **negative** | **AD-P** | 2 (3.9) | 90 (87.4) | 34 (29.1) | 14 (27.5) | 1 (12.5) | 2 (28.6) | 0 (0.0) | 7 (63.6) | 1 (25.0) | 9 (25.0) | 1 (50.0) | 2 (15.4) |
|  | **FT-P** | 10 (19.6) | 13 (12.6) | 68 (58.1) | 34 (66.7) | 6 (75.0) | 5 (71.4) | 0 (0.0) | 1 (9.1) | 2 (50.0) | 20 (55.6) | 1 (50.0) | 3 (23.1) |
|  | **N-P** | 3 (5.9) | 0 (0.0) | 15 (12.8) | 3 (5.9) | 1 (12.5.0) | 0 (0.0) | 0 (0.0) | 3 (27.3) | 1 (25.0) | 7 (19.4) | 0 (0.0) | 6 (46.2) |
|  | **Normal** | 36 (70.6) | 0 (0.0) | 0 (0.0) | 0 (0.0) | 0 (0.0) | 0 (0.0) | 0 (0.0) | 0 (0.0) | 0 (0.0) | 0 (0.0) | 0 (0.0) | 2 (15.4) |
| Aβ  **Positive** | **AD-P** | 11 (61.1) | 515 (80.7) | 11 (55.0) | 5 (71.4) | 1 (50.0) | 1 (50.0) | 1 (50.0) | 3 (60.0) | 0 (0.0) | 0 (0.0) | 10 (90.9) | 1 (50.0) |
|  | **FT-P** | 3 (16.7) | 91 (14.3) | 8 (40.0) | 2 (28.6) | 1 (50.0) | 1 (50.0) | 1 (50.0) | 1 (20.0) | 1 (100.0) | 1 (100.0) | 1 (9.1) | 1 (50.0) |
|  | **N-P** | 3 (16.7) | 28 (4.4) | 1 (5.0) | 0 (0.0) | 0 (0.0) | 0 (0.0) | 0 (0.0) | 1 (20.0) | 0 (0.0) | 0 (0.0) | 0 (0.0) | 0 (0.0) |
|  | **Normal** | 1 (5.6) | 4 (0.6) | 0 (0.0) | 0 (0.0) | 0 (0.0) | 0 (0.0) | 0 (0.0) | 0 (0.0) | 0 (0.0) | 0 (0.0) | 0 (0.0) | 0 (0.0) |
| **Tau**  **Negative** | **AD-P** | 5 (50.0) | 10 (90.9) | 0 (0.0) | 0 (0.0) | 0 (0.0) | 0 (0.0) | 0 (0.0) | 0 (0.0) | 0 (0.0) | 0 (0.0) | 0 (0.0) | 1 (33.3) |
|  | **FT-P** | 2 (20.0) | 1 (9.1) | 2 (100.0) | 0 (0.0) | 0 (0.0) | 0 (0.0) | 0 (0.0) | 1 (100.0) | 0 (0.0) | 1 (100.0) | 0 (0.0) | 1 (33.3) |
|  | **N-P** | 1 (10.0) | 0 (0.0) | 0 (0.0) | 0 (0.0) | 0 (0.0) | 0 (0.0) | 0 (0.0) | 0 (0.0) | 0 (0.0) | 0 (0.0) | 1 (100.0) | 1 (33.3) |
|  | **Normal** | 2 (20.0) | 0 (0.0) | 0 (0.0) | 0 (0.0) | 0 (0.0) | 0 (0.0) | 0 (0.0) | 0 (0.0) | 0 (0.0) | 0 (0.0) | 0 (0.0) | 0 (0.0) |
| **Tau**  **Positive** | **AD-P** | 1 (50.0) | 343 (95.5) | 45 (33.3) | 19 (32.8) | 2 (20.0) | 3 (33.3) | 1 (50.0) | 10 (66.7) | 1 (20.0) | 9 (25.0) | 9 (75.0) | 2 (40.0) |
|  | **FT-P** | 1 (50.0) | 14 (3.9) | 74 (54.8) | 36 (62.1) | 7 (70.0) | 6 (66.7) | 1 (50.0) | 1 (6.7) | 3 (60.0) | 20 (55.6) | 1 (8.3) | 3 (60.0) |
|  | **N-P** | 0 (0.0) | 2 (0.6) | 16 (11.9) | 3 (5.2) | 1 (10.0) | 0 (0.0) | 0 (0.0) | 4 (26.7) | 1 (20.0) | 7 (19.4) | 2 (16.7) | 0 (0.0) |
|  | **Normal** | 0 (0.0) | 0 (0.0) | 0 (0.0) | 0 (0.0) | 0 (0.0) | 0 (0.0) | 0 (0.0) | 0 (0.0) | 0 (0.0) | 0 (0.0) | 0 (0.0) | 0 (0.0) |
| **APOE ε4**  **non-carriers** | **AD-P** | 3 (42.9) | 52 (94.5) | 19 (33.9) | 8 (33.3) | 1 (20.0) | 1 (33.3) | 1 (50.0) | 6 (60.0) | 2 (66.7) | 2 (22.2) | 11 (78.6) | 3 (25.0) |
|  | **FT-P** | 1 (14.3) | 1 (1.8) | 29 (51.8) | 16 (66.7) | 3 (60.0) | 2 (66.7) | 1 (50.0) | 1 (10.0) | 1 (33.3) | 4 (44.4) | 1 (7.1) | 7 (58.3) |
|  | **N-P** | 2 (28.6) | 1 (1.8) | 8 (14.3) | 0 (0.0) | 1 (20.0) | 0 (0.0) | 0 (0.0) | 3 (30.0) | 0 (0.0) | 3 (33.3) | 1 (7.1) | 2 (16.7) |
|  | **Normal** | 1 (14.3) | 1 (1.8) | 0 (0.0) | 0 (0.0) | 0 (0.0) | 0 (0.0) | 0 (0.0) | 0 (0.0) | 0 (0.0) | 0 (0.0) | 1 (7.1) | 0 (0.0) |
| **APOE ε4**  **carrier** | **AD-P** | 4 (80.0) | 43 (79.6) | 4 (30.8) | 3 (30.0) | 0 (0.0) | 0 (0.0) | 0 (0.0) | 1 (100.0) | 0 (0.0) | 0 (0.0) | 1 (100.0) | 1 (33.3) |
|  | **FT-P** | 1 (20.0) | 7 (13.0) | 7 (53.8) | 5 (50.0) | 0 (0.0) | 1 (100.0) | 0 (0.0) | 0 (0.0) | 1 (100.0)) | 0 (0.0) | 0 (0.0) | 2 (66.7) |
|  | **N-P** | 0 (0.0) | 3 (5.6) | 2 (15.4) | 2 (20.0) | 0 (0.0) | 0 (0.0) | 0 (0.0) | 0 (0.0) | 0 (0.0) | 0 (0.0) | 0 (0.0) | 0 (0.0) |
|  | **Normal** | 0 (0.0) | 1 (1.9) | 0 (0.0) | 0 (0.0) | 0 (0.0) | 0 (0.0) | 0 (0.0) | 0 (0.0) | 0 (0.0) | 0 (0.0) | 0 (0.0) | 0 (0.0) |

Abbreviations: Aβ, Amyloid-β; MCI, mild cognitive impairment; AD, Alzheimer’s disease; FTLD, frontotemporal lobar degeneration; DLB, dementia with Lewy bodies; VaD, vascular dementia; ODs, other diagnosis; bvFTD, behavioral variant frontotemporal dementia; SD, semantic dementia; PNFA, progressive non-fluent aphasia; PPA, Primary progressive aphasia; CBD, cortical baseal degeneration; FTLD/ALS, frontotemporal lobar degeneration and amyotrophic lateral sclerosis; PSP, progressive supranuclear palsy; AD-P, AD pattern hypometabolism; FT-P, frontotemporal lobe-pattern hypometabolism; N-P, non-specific hypometabolism; APOE ε4, Apolipoprotein ε4.
